# Supplementary material for: Orbitofrontal cortex contributes to the comparison of values underlying economic choices
Source: Nat Commun. 2022 Jul 29;13:4405. doi: 10.1038/s41467-022-32199-y (PMC9338286; doi:10.1038/s41467-022-32199-y)
Supplement: Supplementary file 1 — Supplementary Information [file 41467_2022_32199_MOESM1_ESM.pdf]

Orbitofrontal cortex contributes to the comparison of values underlying economic choices

S. Ballesta, W. Shi and C. Padoa-Schioppa

Supplementary Information

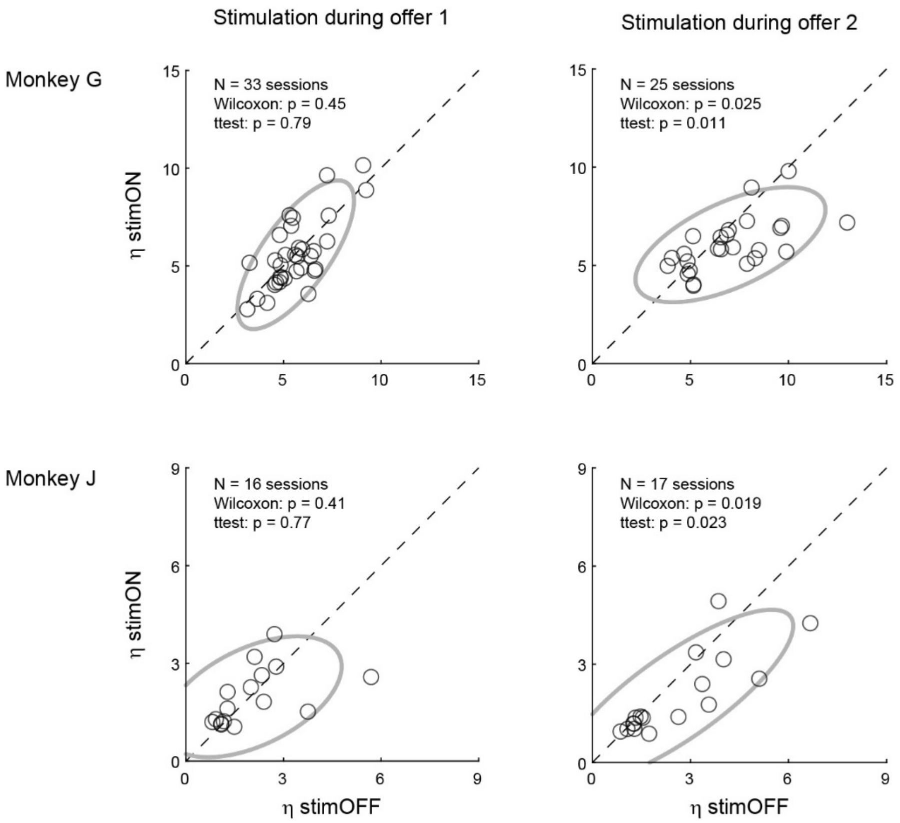

Supplementary Fig.1. Effects of weak OFC stimulation on choice variability, individual animals. The figure summarizes the changes in choice variability measured upon stimulation at 5-15 μA during offer1 and offer2, for each animal. In each panel, x-axis and y-axis represent the sigmoid steepness measured for stimOFF and stimON trials, respectively. Each data point represents one session, the ellipse represents the 90% confidence interval, and results of statistical tests are reported. Stimulation during offer1 did not systematically alter the sigmoid steepness in either animal. In contrast stimulation during offer2 significantly reduced the sigmoid steepness in both animals. All p values were obtained from two-tailed Wilcoxon tests and two-tailed t tests. Source data are provided as a Source Data file.

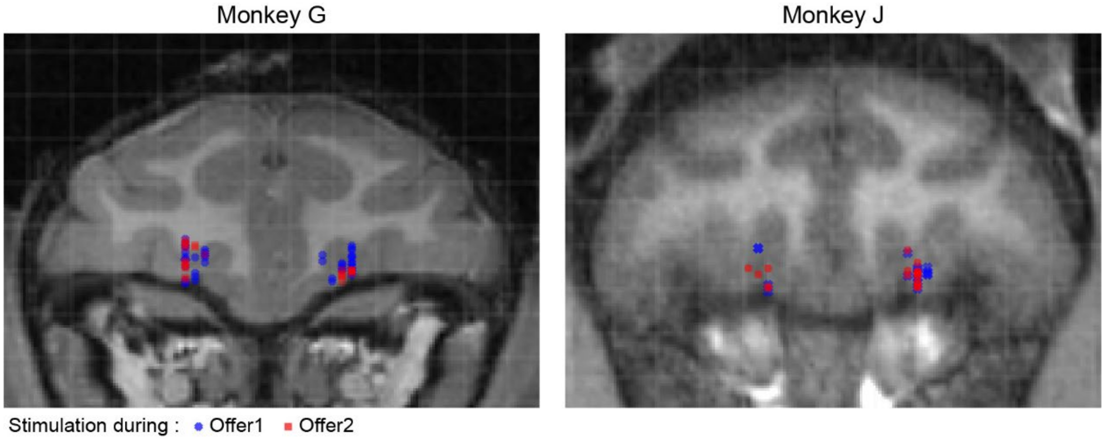

Supplementary Fig.2. Reconstruction of stimulation sites. Red and blue indicate stimulations delivered during offer1 and offer2, respectively. For each animal, stimulation sites ranged on the AP axis between AP31 and AP36. In the figure, the stimulation sites were collapsed on the AP dimension and displayed on the coronal section corresponding to AP33. Source data are provided as a Source Data file.

		**5-15 µA**	**25 µA**	**50 µA**	**125 µA**
Range-dependent bias (offer1 + offer2)	N sessions<br>mean ± s.e.<br>p value	90<br>0.13 ± 0.10<br>0.21	46<br>0.70 ± 0.22<br>**0.0025**	40<br>1.00 ± 0.21<br>**2.2 10^{-5}**	52<br>0.06 ± 0.23<br>0.78
Change in order bias, offer1	N sessions<br>mean ± s.e.<br>p value	49<br>0.05 ± 0.02<br>0.010	29<br>-0.07 ± 0.08<br>0.46	22<br>0.39 ± 0.08<br>**5.5 10^{-4}**	29<br>0.72 ± 0.13<br>**8.8 10^{-6}**
Change in order bias, offer2	N sessions<br>mean ± s.e.<br>p value	42<br>-0.02 ± 0.04<br>0.35	17<br>0.03 ± 0.18<br>0.69	22<br>0.62 ± 0.18<br>**0.0041**	25<br>1.00 ± 0.24<br>**3.0 10^{-4}**
Change in choice variability, offer1	N sessions<br>mean ± s.e.<br>p value	49<br>0.07 ± 0.17<br>0.59	29<br>0.22 ± 0.24<br>0.47	22<br>-0.39 ± 0.26<br>0.20	29<br>0.04 ± 0.38<br>0.84
Change in choice variability, offer2	N sessions<br>mean ± s.e.<br>p value	42<br>0.82 ± 0.23<br>**0.0011**	17<br>-0.48 ± 0.44<br>0.27	22<br>0.34 ± 0.26<br>0.10	25<br>0.68 ± 0.19<br>**0.0025**

Supplementary Table 1. Normalized effect sizes and exact p values across conditions. Mean and s.e. were rectified and normalized to the maximal effect across conditions (see **Methods**). For the range-dependent bias, p values are from Pearson's correlation. All other p values are from two-tailed Wilcoxon tests. Bold highlights p values <0.01.
