# Supplementary material for: Orbitofrontal cortex contributes to the comparison of values underlying economic choices
Source: Nat Commun. 2022 Jul 29;13:4405. doi: 10.1038/s41467-022-32199-y (PMC9338286; doi:10.1038/s41467-022-32199-y)
Supplement: Supplementary file 3 — Reporting Summary [file 41467_2022_32199_MOESM3_ESM.pdf]

nature portfolio

Corresponding author(s): Padoa-Schioppa, Camillo

Last updated by author(s): Jun 7, 2022

Reporting Summary

Nature Portfolio wishes to improve the reproducibility of the work that we publish. This form provides structure for consistency and transparency in reporting. For further information on Nature Portfolio policies, see our Editorial Policies and the Editorial Policy Checklist.

Statistics

For all statistical analyses, confirm that the following items are present in the figure legend, table legend, main text, or Methods section.

n/a	Confirmed	
☐	☒	The exact sample size (*n*) for each experimental group/condition, given as a discrete number and unit of measurement
☒	☐	A statement on whether measurements were taken from distinct samples or whether the same sample was measured repeatedly
☐	☒	The statistical test(s) used AND whether they are one- or two-sided<br>*Only common tests should be described solely by name; describe more complex techniques in the Methods section.*
☐	☒	A description of all covariates tested
☐	☒	A description of any assumptions or corrections, such as tests of normality and adjustment for multiple comparisons
☐	☒	A full description of the statistical parameters including central tendency (e.g. means) or other basic estimates (e.g. regression coefficient) AND variation (e.g. standard deviation) or associated estimates of uncertainty (e.g. confidence intervals)
☐	☒	For null hypothesis testing, the test statistic (e.g. *F*, *t*, *r*) with confidence intervals, effect sizes, degrees of freedom and *P* value noted<br>*Give P values as exact values whenever suitable.*
☒	☐	For Bayesian analysis, information on the choice of priors and Markov chain Monte Carlo settings
☒	☐	For hierarchical and complex designs, identification of the appropriate level for tests and full reporting of outcomes
☐	☒	Estimates of effect sizes (e.g. Cohen's *d*, Pearson's *r*), indicating how they were calculated

Our web collection on statistics for biologists contains articles on many of the points above.

Software and code

Policy information about availability of computer code

Data collection	Behavior was controlled through a custom-written software (monkeylogic) based on Matlab (http://www.monkeylogic.net/). Data were collected in Matlab (v 2016b).
Data analysis	Data were analyzed in Matlab (v 2016b). Analysis codes are available at: https://github.com/PadoaSchioppaLab/2022_NatComms_lowCurrent

For manuscripts utilizing custom algorithms or software that are central to the research but not yet described in published literature, software must be made available to editors and reviewers. We strongly encourage code deposition in a community repository (e.g. GitHub). See the Nature Portfolio guidelines for submitting code & software for further information.

Data

Policy information about availability of data

All manuscripts must include a data availability statement. This statement should provide the following information, where applicable:

- Accession codes, unique identifiers, or web links for publicly available datasets
- A description of any restrictions on data availability
- For clinical datasets or third party data, please ensure that the statement adheres to our policy

The raw data and the Matlab code used for the analysis are available at https://github.com/PadoaSchioppaLab/2022_NatComms_lowCurrent

Field-specific reporting

Please select the one below that is the best fit for your research. If you are not sure, read the appropriate sections before making your selection.

☒ Life sciences ☐ Behavioural & social sciences ☐ Ecological, evolutionary & environmental sciences

For a reference copy of the document with all sections, see nature.com/documents/nr-reporting-summary-flat.pdf

Life sciences study design

All studies must disclose on these points even when the disclosure is negative.

Sample size	We did not pre-determine the number of sessions per condition. We aimed to ~20 sessions per monkey in each condition. These numbers are above current standards for the field, and similar with the ones in our previous study Ballesta et al. Nature 2020.
Data exclusions	In all the analyses, we identified as outliers data points that differed from the mean by >3 STD on either axis, and we removed them from the data set. In the primary analyses, there were no outliers. In the analyses of range-dependent biases, the criterion excluded 1/91 sessions.
Replication	The results were replicated and held true in each of the two monkeys.
Randomization	For each monkey, we randomized the sessions with offer1 stimulation and offer2 stimulation. For each session, we randomized the trials with stimulation and trials without stimulation.
Blinding	Group allocation does not apply to our study

Reporting for specific materials, systems and methods

We require information from authors about some types of materials, experimental systems and methods used in many studies. Here, indicate whether each material, system or method listed is relevant to your study. If you are not sure if a list item applies to your research, read the appropriate section before selecting a response.

Materials & experimental systems

n/a	Involved in the study	
☒	☐	Antibodies
☒	☐	Eukaryotic cell lines
☒	☐	Palaeontology and archaeology
☐	☒	Animals and other organisms
☒	☐	Human research participants
☒	☐	Clinical data
☒	☐	Dual use research of concern

Methods

n/a	Involved in the study	
☒	☐	ChIP-seq
☒	☐	Flow cytometry
☒	☐	MRI-based neuroimaging

Animals and other organisms

Policy information about studies involving animals; ARRIVE guidelines recommended for reporting animal research

Laboratory animals	The study was conducted on two male rhesus monkeys: G (age 8, 9.1 kg) and J (age 7, 10.0 kg).
Wild animals	The study did not involve wild animals.
Field-collected samples	The study did not involve samples from the field.
Ethics oversight	All experimental procedures conformed to the NIH Guide for the Care and Use of Laboratory Animals and were approved by the Institutional Animal Care and Use Committee (IACUC) at Washington University.

Note that full information on the approval of the study protocol must also be provided in the manuscript.
